# Ultraviolet A irradiation induces senescence in human dermal fibroblasts by down-regulating DNMT1 via ZEB1

**DOI:** 10.18632/aging.101383

**Published:** 2018-02-16

**Authors:** Yuxin Yi, Hongfu Xie, Xiao Xiao, Ben Wang, Rui Du, Yingzi Liu, Zibo Li, Jun Wang, Lunquan Sun, Zhili Deng, Ji Li

**Affiliations:** 1 Department of Dermatology, Xiangya Hospital, Central South University, Changsha, China; 2 Key Laboratory of Organ Injury, Aging and Regenerative Medicine of Hunan Province, China; 3 Center for Molecular Medicine, Xiangya Hospital, Central South University, Changsha, China; 4 The State Key Laboratory of Medical Genetics & School of Life Sciences, Central South University, Changsha, China; 5 National Clinical Research Center for Geriatric Disorders, Changsha, China

**Keywords:** UVA, ZEB1, DNMT1, methylation, senescence

## Abstract

In this study, we report the role of DNA methyltransferase 1 (DNMT1) in ultraviolet A (UVA)-induced senescence in human dermal fibroblasts (HDFs). We show that DNMT1 expression was significantly reduced during UVA-induced senescence, and this senescence could be alleviated or aggravated by the up- or down-regulation of DNMT1, respectively. Expression of the transcription factor zinc finger E-box binding homeobox 1(ZEB1) also decreased after UVA irradiation, following a UVA-induced increase of intracellular reactive oxygen species (ROS). We show that ZEB1 binds to the DMNT1 promoter and regulates its transcription, which, in turn, affects cellular senescence. These changes in DMNT1 and ZEB1 expression following UVA exposure were confirmed in matched skin specimens that had or had not been sun-exposed. On analyzing the promoter methylation of 24 senescence associated genes in these matched skin specimens, we discovered that p53 promoter methylation was significantly reduced in sun-exposed skin. *In vitro* experiments confirmed that UVA irradiation reduced p53 promoter methylation, and DNMT1 up-regulation could reverse this effect. Collectively, down-regulation of ZEB1 caused by UVA induced ROS could transcriptionally inhibit DNMT1, leading to low methylation level of senescence related proteins p53 and increase its expression, eventually result in cellar senescence.

## Introduction

Chronic exposure to solar ultraviolet (UV) radiation is a major cause of premature skin aging, also known as photoaging. Among three kinds of UV (UVA, UVB, UVC), UVA has been proved to be responsible for most of the chronic skin damage associated with photoaging, due to its abundance and deep penetration into the dermis [1-3]. UV induces responses such as oxidative stress, inflammatory responses, DNA damage and immune suppression, significantly affects epigenetic regulation, thus altering the gene expression programs that contribute to aging. Epigenetic features such as DNA methylation patterns and histone modifications are altered by exposure to UV radiation [4,5]. Indeed, sun exposure induced an epigenetic shift towards DNA hypomethylation in human skin, and the degree of hypomethylation correlated with clinical photoaging measures [6]. Furthermore, increased H3K4me3 modifications and decreased H3K9me2 modifications, as well as a concomitant increase in matrix metalloproteinase(MMP)-1 and MMP3 mRNA levels, were observed in HDFs irradiated with solar-simulated UVR [7]. Additionally, increases in histone H3-K9/14 acetylation within the promoter regions of ATF3, COX2, IL-8, MKP1, and MnSOD have been reported in UV-irradiated human keratinocytes [8].

Low-level DNA methylation is associated with photoaging, but there is little evidence that DNMT1, the methyltransferase primarily responsible for maintaining genomic methylation stability, is important in UV-induced photoaging. However, there is some evidence linking DNMT1 with aging and/or senescence in the context of other etiological factors or circumstances. For example, RNA interference (RNAi) knockdown of DNMT1 in middle-aged 2BS and WI-38 fibroblasts resulted in up-regulation of p21(Waf1/Cip1) and premature senescence [9]. Similarly, small interfering RNA (siRNA) knockdown of DNMT1 induced cellular senescence in human umbilical cord blood-derived multi-potent stem cells, and increased p16(INK4A) and p21(CIP1/WAF1) expression [10]. Furthermore, DNMT1 knockdown in early-passage mesenchymal stromal cells (MSCs) induced senescence and reduced differentiation potential, whereas DNMT1 over-expression in late-passage MSCs had the opposite effect [11]. Another study also proved in vivo that with epidermal deletion of DNMT1, skin stem cell activation probability decreased during aging of mice [12]. In our preliminary experiments, we showed, for the first time, that DNMT1 expression was significantly decreased in HDFs following UVA irradiation. We therefore speculated that DNMT1 might play a key role in skin photoaging that has not been reported to date.

Presently, the expression and activity of DNMT1 are known to be regulated via transcriptional regulation, post-transcriptional controls, and post-translational modifications. For example, the transcription factors activator protein 1(AP-1), E2F, protein 53 (p53), and specificity protein 1 (SP1) all bind to the DNMT1 promoter in order to transcriptionally modify its expression [13].Additionally, post-transcriptional gene silencing by microRNAs, including miR-152, miR-185, miR-126 and miR-377, which directly interact with the3'-UTR of DMNT1 mRNA, could be important in the regulation of DMNT1 expression [14-17]. Alternatively, the expression and activity of DNMT1 could be affected indirectly by microRNAs such as miR-28b and miR-290, which affect interacting transcription factors such as SP1 and Rb [18,19]. Other research has shown that reversible covalent post-translational DNMT1 modifications, including methylation, acetylation, phosphorylation, sumoylation, and ubiquitination, can greatly affect its activity, stability and interactions with other proteins [20]. However, the regulatory mechanisms underlying the control of DNMT1 expression and activity in HDFs following UVA irradiation remain poorly understood. We performed bioinformatics analysis using the JASPAR database, and predicted four high-scoring binding sites for the transcription factor zinc finger E-box-binding homeobox 1 (ZEB1) in the DNMT1 promoter. Fukagawa A and colleagues proved that ZEB1 interacted with DNMT1 through the Smad-binding domain, promoted methylation of E-cadherin in breast cancer cells [21]. Interestingly, ZEB1 expression is often suppressed by elevated ROS levels [22,23], which can themselves be produced by UVA irradiation. Furthermore, ZEB1 is associated with aging, and ZEB1 down-regulation plays a key role in ROS-induced senescence [23]. Additionally, ZEB1 RNAi knockdown resulted in the induction of p15 (INK4B) and p16 (INK4A), and reactivation of the EGFR-dependent senescence program [24]. ZEB1 is therefore a promising candidate for DNMT1 regulation following UVA irradiation, and may play a vital role in photoaging.

We therefore used HSFs to study the role of DNMT1 in UVA-induced senescence. We found that UVA irradiation reduced DNMT1 expression; while recovery of this expression attenuated UVA-induced senescence, DNMT1 suppression provoked the opposite effect. Additionally, DNMT1-mediated prevention of senescence was associated with the restoration of methylation of the senescence-associated gene p53. ZEB1 expression decreased following UVA irradiation, which could be due to increased intracellular ROS production under these conditions. ZEB1 was found to directly bind to the DNMT1 promoter in order to regulate its expression, thus affecting UVA induced senescence. At last, we found the identical changes ofDNMT1 and ZEB1 expression in vivo.

In summary, we have described the regulatory relationship between DNMT1 and ZEB1 expression, and have proposed a novel regulatory axis, termed the UVA/ROS/ZEB1/DNMT1/p53 axis, to describe the elements involved in UVA-induced cellular senescence and photoaging.

## RESULTS

### DNMT1 attenuates UVA-induced senescence in HDFs

**Figure 1:**
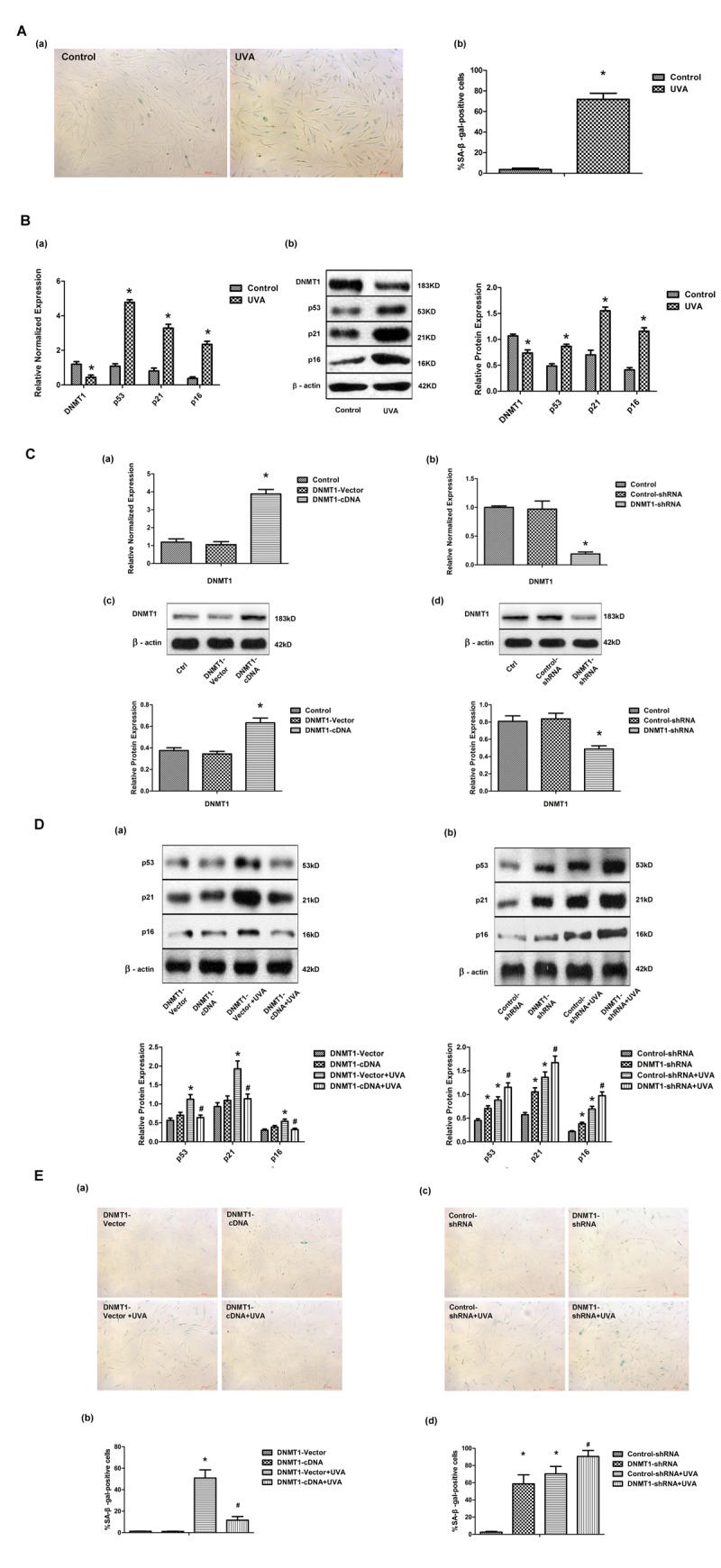
**DNMT1 attenuates UVA-induced senescence in HDFs.** (**A**)Senescence-associated β-galactosidase (SA-β-gal) activity in HDFs, showing representative images from three independent experiments (a), (scale bar = 200 µm), and the mean percentage of SA-β-gal-positive cells (b).Error bars represent standard deviation from the mean.* vs control, P < 0.05. (**B**). (a) DNMT1, p53, p21, and p16 mRNA expression, as determined by real-time PCR. Each sample was analyzed in triplicate for each condition. Data are shown as the mean of three independent experiments. * vs control, P < 0.05. (b) DNMT1, p53, p21, and p16 protein expression, as determined by Western blot analysis (left panels). Bar graphs (right panels) show quantitative analysis of scanning densitometric values of these proteins as ratios to β-actin, which was used as a loading control. Data are representative of three independent experiments. * vs control, P < 0.05. (**C**) DNMT1 expression at the mRNA level (a, b) and the protein level (c, d), determined by real-time PCR or Western blotting, respectively, in HDFs transfected with either DNMT-cDNA or DNMT-shRNA expressing lentivirus (n = 3).* vs DNMT-vector or control-shRNA, P< 0.05. (**D**)Western blots images (upper panels) and quantitative analysis (lower panels) showing p53, p21, and p16 protein expression. Data are epresentative of three independent experiments. (**E**)Senescence-associated β-galactosidase(SA-β-gal) activity in cells under the indicated conditions. Representative images are shown (scale bar = 200 µm). The percentages of SA-β-galpositive cells under each condition are presented as the mean ± standard deviation of three independent experiments. * vs DNMT-vector or control-shRNA, P < 0.05;# vs DNMT1-vector+UVA or control-shRNA+UVA, P < 0.05.

Primary HDFs were irradiated with 10J/cm^2^ UVA per day for 3 days, and then cellular senescence was evaluated by measuring senescence-associated β-galactosidase (SA-β-gal) activity. The percentage of cells that were SA-β-gal positive, and thus senescent, was significantly higher in UVA-irradiated HDFs than in controls (Fig. 1A). Consistent with this, the expression of the senescence markers p53, p21, and p16, as shown by real-time PCR and Western blot, was significantly increased after UVA irradiation (Fig. 1B). These data suggested that UVA irradiation caused HDFs to enter a senescence-like state. Interestingly, DNMT1expression in HDFs was markedly down-regulated at both the mRNA and protein levels following UVA irradiation (Fig. 1B). Next, the role of DNMT1 in UVA-induced senescence was investigated by stably transfecting HDFs with DNMT-cDNA and DNMT-shRNA expressing lentiviral vectors in order to enhance or inhibit DNMT expression, respectively. Modifications to DNMT1 expression were confirmed using real-time PCR and Western blotting (Fig.1C). Cellular senescence in the presence of altered DNMT1 expression was then evaluated by measuring SA-β-gal activity and p53, p21, and p16 expression. Up-regulation of DNMT1 was found to partially reverse the observed UVA-induced increase in SA-β-gal positive cells. Similarly, DNMT1 expression attenuated the observed increase in p53, p21, and p16 expression following UVA irradiation (Fig. 1D (a), and E (a, b)). Furthermore, DNMT1 down-regulation, in the presence or absence of UVA irradiation, led to higher numbers of SA-β-gal positive cells and higher expression of senescence-related proteins (Fig. 1D (b), and E (c, d)).

### ZEB1 attenuates UVA-induced senescence in HDFs via DNMT1

**Figure 2:**
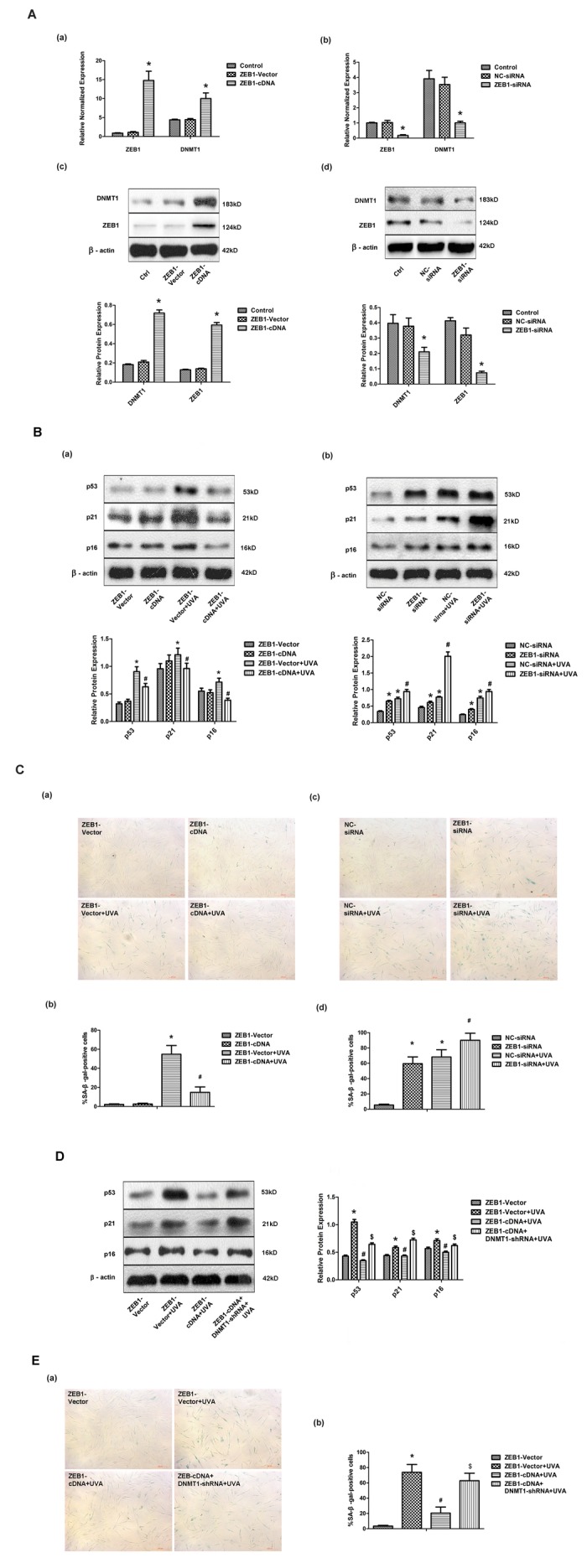
**ZEB1 attenuates UVA-induced senescence in HDFs via DNMT1.** (**A**) ZEB1 and DNMT1 expression in HDFs at the mRNA (a, b) and protein (c, d) levels following ZEB1 over-expression or knockdown, as determined by real-time PCR and Western blotting, respectively (n = 3).* vs ZEB1-vector or negative control (NC)-siRNA, P< 0.05. (**B**) Western blots images (upper panels) and quantitative analysis (lower panels), representative of three independent experiments, were showing p53, p21, and p16 protein expression. (**C**) Senescence-associated β-galactosidase(SA-β-gal) activity of cells under the indicated conditions. Representative images are shown (scale bar = 200 µm). The percentages of SA-β-galpositive cells under each condition are presented as the mean ± standard deviation of three independent experiments. * vs ZEB1-vector or NC-siRNA, P < 0.05;# vs ZEB1-vector+UVA or NC-siRNA+UVA, P < 0.05. (**D**) Western blots images (left panels) and quantitative analysis (right panels), representative of three independent experiments, showing p53, p21, and p16 protein expression in HDFs co-transfected with ZEB1-cDNA and DNMT1-shRNA. (**E**) SA-β-gal activity of cells under the indicated conditions, following DNMT1 knockdown. Cells were analyzed as described in (**C**). * vs ZEB1-vector, P < 0.05;# vs ZEB1-vector+UVA, P < 0.05; $ vs ZEB1-cDNA+UVA.

Having shown that UVA irradiation modulated DNMT1 expression at the transcriptional level, we investigated the involvement of other factors in this process. First, we analyzed the DNMT1 promoter sequence using the bioinformatics tool JASPAR, and discovered multiple, high-scoring predicted binding sites for the transcription factor ZEB1. We confirmed that ZEB1 regulated DNMT1 expression in HDFs by down- or up- regulating ZEB1 expression with a ZEB1-siRNA or ZEB1-cDNA expressing lentiviral vector, respectively (Fig. 2A). Additionally, ZEB1 up-regulation partially reversed the UVA-induced increase in SA-β-gal positive cells and up-regulation of p53, p21, and p16 expression, while ZEB1 down-regulation caused the opposite effects to be observed (Fig. 2B and C). shRNA knockdown of DNMT1 expression blocked the effects of ZEB1 up-regulation (Fig. 2D and E). Taken together, these data suggest that ZEB1-mediated effects on cellular senescence following UVA irradiation occur via DNMT1.

### ZEB1 binds directly to the DNMT1 promoter and regulates its transcription

**Figure 3:**
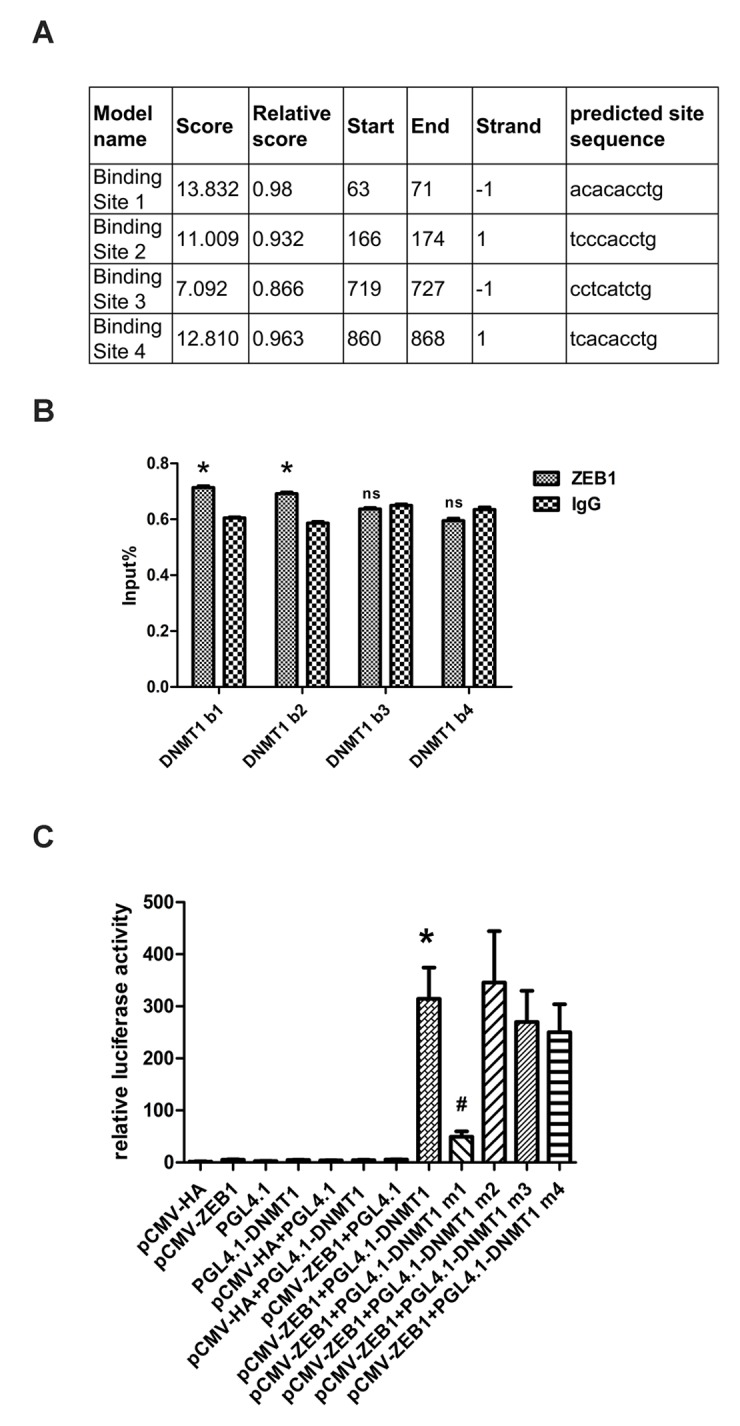
**ZEB1 binds directly to the DNMT1 promoter and regulates its transcription.** (**A**) Schematic showing the region of the DNMT1 promoter containing potential ZEB1 binding sites. (**B**) Chromatin immunoprecipitation data from HDFs incubated with either anti-ZEB1antibody or non-specific control IgG, showing the amplification of each of the four predicted ZEB1 binding sites within the DNMT1 promoter (termed DNMT1 b1, 2, 3, and 4). Experiments were performed in triplicate.* vs IgG, P < 0.05;ns vs IgG, P>0.05. (**C**) Luciferase reporter assay data, showing the activity of either the wild type (WT)DNMT1 promoter or mutants lacking each of the predicted ZEB1 binding sites. Cells were transfected with the following plasmids: ZEB1: ZEB1-cDNA-expressing vector; WT DNMT1: reporter plasmid containing WT DNMT1 promoter; DNMT1 Mut1-4: reporter plasmids containing the DNMT1 promoter with putative ZEB1 binding sites 1-4 deleted. Experiments were performed in triplicate. * vs pCWV-ZEB1+pGL4.1, P<0.05; # vs pCWV-ZEB1+pGL4.1-DNMT1, P <0.05.

Having shown that ZEB1 regulates DNMT1 expression, we investigated the underlying regulatory mechanisms by cloning the DNMT1 promoter region (positions -1000bp to +194bp), which contains four high-scoring predicted ZEB1 binding sites (Fig. 3A). The binding of ZEB1 to these sequences was then measured using chromatin immunoprecipitation (ChIP) assays. The sequences at predicted binding sites 1 and 2 were amplified to a greater extent following immunoprecipitation with an anti-ZEB1 antibody than with the non-specific IgG control (Fig. 3B), suggesting that ZEB1 binds to predicted sequences 1 and 2. To confirm that this binding enhanced DNMT1 promoter activity, we generated luciferase reporter vectors containing either the wild type (WT) DNMT1 promoter sequence or 4 deletion mutants that lacked the predicted ZEB1 binding sites 1-4, which we termed Mut1-4, respectively. Next, we cotransfected HEK293T cells with either ZEB1-cDNA expressing vector or control vector alone with each reporter vector. Compared with the control vector, ZEB1 over-expression caused a significant increase in luciferase activity in the cells that were cotransfected with the WT and Mut2-4 reporter vectors. Conversely, cotransfection of ZEB1-expressing vector with the Mut1 reporter vector gave a level of luciferase activity that was equivalent to the control (Fig. 3C). Thus we determined that predicted ZEB1 binding site 1 was essential for regulation of DNMT1 promoter activity. While ZEB1 was shown to bind to predicted binding site 2 in ChIP assays, its removal did not prevent an increase in luciferase reporter activity; indeed, this increase was slightly greater than was seen with the wild type in dual reporter assays, although this difference was not statistically significant. As ZEB1 can transcriptionally activate or suppress target genes, it is possible that binding site 2 allows it to negatively regulate DNMT1 promoter activity.

### UVA irradiation regulates transcription factor ZEB1 via ROS

**Figure 4:**
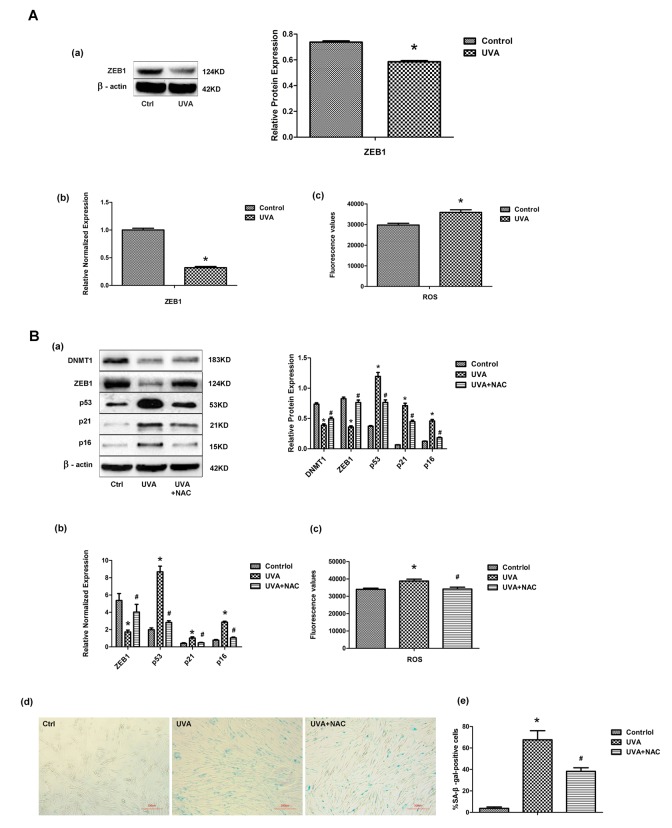
**UVA irradiation regulates transcription factor ZEB1 via ROS.** (**A**) ZEB1 mRNA (a) and protein (b) expression, assessed by real-time PCR and Western blotting, respectively, in irradiated and non-irradiated HDFs. Experiments were performed in triplicate. Intracellular ROS levels were assessed by measuring dichlorofluorescein (DCF) in triplicate experiments. * vs control, P < 0.05. (**B**) Following treatment with N-acetyl-L-cysteine (NAC), the expression of ZEB1, p53, p21, and p16 was assessed at the protein (a) and mRNA (b) levels. Experiments were performed in triplicate. * vs control or UVA, P < 0.05; Senescence-associated β-galactosidase (SA-β-gal) activity was assessed to evaluate cellular senescence after NAC treatment. In each condition the number of SA-β-gal-positive cells were counted (c, d; scale bar=200 µm). Experiments were performed in triplicate. * vs control or UVA, P < 0.05; Western blots images (left panels) and quantitative analysis (right panels) are representative of three independent experiments.

Following UVA irradiation of HDFs, ZEB1 mRNA and protein expression dropped significantly, whereas levels of intracellular ROS were markedly increased (Fig. 4A). Previously, it was suggested that intracellular ROS was responsible for the regulation of ZEB1 expression [23]. We therefore used the antioxidant N-acetyl-L-cysteine (NAC) to inhibit the UVA-induced increase in ROS, and showed both that ZEB1 and DNMT1 expression levels were restored and cellular senescence was attenuated (Fig. 4B). These data suggest that UVA irradiation decreases ZEB1 expression through the induction of ROS.

### DNMT1 regulates p53 by modifying CpG methylation

**Figure 5:**
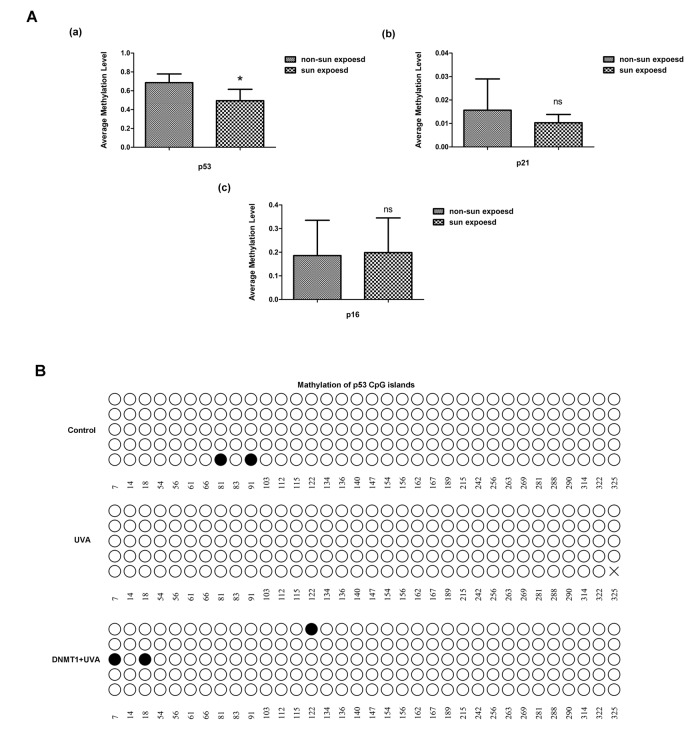
**DNMT1 regulates p53 by modifying CpG methylation**. (**A**) Relative methylation of the promoters of the senescence-associated genes p53, p21, and p16, in sun-exposed or non-sun-exposed human skin samples, * vs non-sun exposed, P < 0.05. (**B**) Relative methylation of the p53 promoter region in untreated HDFs(a), UVA-irradiated HDFs (b), and UVA-irradiated HDFs over-expressing DNMT1(c). Each horizontal line represents an individual DNA molecule, and the circles represent CpG dinucleotides. Filled circles: methylated CpGs; open circles: unmethylated CpGs. Numbers at the bottom of the figure indicates CpG position.

The promoter methylation levels of 24 senescence-associated genes were compared between matched human skin specimens that had or had not been exposed to sun or non-sun exposed (Supplementary Table 4). Methylation of the p53 promoter was significantly lower in sun-exposed skin than in non-sun-exposed samples (Fig. 5A). Similarly, bisulfite sequencing analysis confirmed in HDFs that UVA irradiation caused demethylation of CpG in the p53 promoter region, while DNMT1 up-regulation could methylate CpG islands of p53, although the demethylation caused by UVA and DNMT1 induced methylation did not occurred at the same CpG islands (Fig.5B). Conversely, there were no significant changes in CpG methylation in the p16 and p21 genes (data not shown). Overall, we have precisely defined the patterns of CpG methylation within the p53 promoter that are modified by DNMT1 during UVA-induced senescence.

### DNMT1 and ZEB1 expression is reduced in sun-exposed human skin

**Figure 6:**
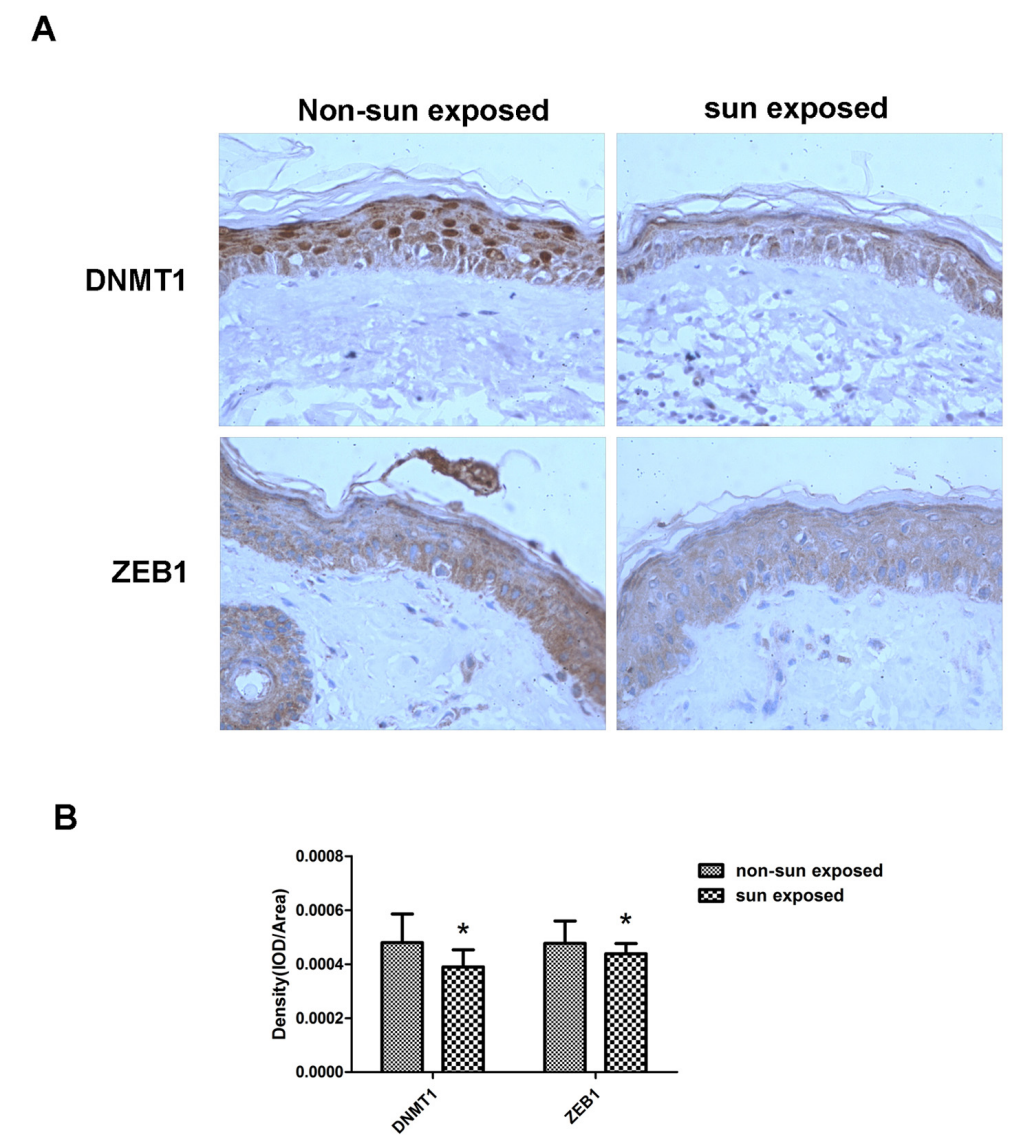
**DNMT1 and ZEB1 expression is reduced in sun-exposed human skin.** (**A**)Representative immunohistochemical staining images of matched human sun-exposed or non-sun-exposed skin specimens from within the same individual. Sections were imaged using primary antibodies raised against DNMT1 or ZEB1 (**C**, **D**) (Magnification= 400×). (**B**) Bar graph shows quantitative analysis of scanning density values of these proteins, * vs non-sun exposed, P < 0.05.

Finally, matched sun-exposed and non-sun-exposed human skin specimens, taken from the same patients, were examined immunohistochemically for DNMT1 and ZEB1 expression. There was a marked decrease in both DNMT1 and ZEB1 expression in the sun-exposed samples, confirming that the *in vitro* changes in expression described in the preceding sections also occurred *in vivo* (Fig. 6).

## DISCUSSION

In the present study, we have shown that DNMT1 attenuated UVA-induced cellular senescence in HDFs by maintaining p53 promoter methylation, thus suppressing its expression. Furthermore, we have shown that DMNT1 expression is regulated by the transcription factor ZEB1, whose expression in turn is regulated by the ROS generated in response to UVA irradiation. We therefore propose a novel UVA/ROS/ZEB1/DNMT1/p53 regulatory axis, which explains the role of DNMT1 in photoaging.

Recently, it was reported that both global DNA hypomethylation and regional hypermethylation occur in aging [25-27], suggesting that dysregulated methylation processes contribute to the initiation and progression of aging. However, the main focus of research into the epigenetic changes that occur following UV-irradiation is photocarcinogenesis rather than photoaging. Several studies have suggested that DNA hypermethylation occurs in UVB-exposed epidermal skin and keratinocytes, and the expression alterations of oncogenes induced by UVB were associated with the elevated expression and activity of DNMT1, DNMT3a and DNMT3b [28-30]. Conversely, we found that UVA irradiation decreased DNMT1expression, and that up- or down-regulation of DNMT1 expression reversed or accelerated cellular senescence, respectively. These seemingly contradictory results can be reconciled by considering that senescence is a well-known protective mechanism against carcinogenesis [31], and that senescence-related alterations in gene expression would be expected to be anti-carcinogenic.

Having defined the role of DNMT1 in UVA-induced senescence, we then identified a regulatory role for the transcription factor ZEB1, which not only influenced UVA-induced senescence, but also modified DNMT1 expression. ZEB1 belongs to the ZEB family of transcription factors, which are characterized by the presence of two zinc finger clusters, through which ZEB1 specifically binds to DNA motifs termed E-boxes. Previously, the DNMT1 gene sequence was found to contain one major and three minor transcription initiation sites (P1–P4) that are regulated by independent enhancer and promoter sequences [20]. Using ChIP analysis and dual-luciferase reporter assays, we proved that ZEB1 bound to the DMNT1 promoter at a sequence we predicted bioinformatically, and regulated DMNT1 transcription. By binding to E-boxes, ZEB1 can recruit co-suppressors or co-activators through its Smad interaction domain, its CtBP interaction domain, orits p300-P/CAF binding domain, to either down-regulate or up-regulate target gene expression [32-35]. For example, ZEB1 binds directly to the CDH1 promoter and recruits the CtBP transcriptional co-repressor, leading to transcriptional repression [36,37]. Conversely, ZEB1 can activate the transcription of TGF-β-responsive genes by recruitingp300-P/CAF and Smad, leading to advanced osteoblastic differentiation [32,33]. In our study, ZEB1was an activator of DNMT1, but the specific underlying mechanisms require further study.

ZEB1 is very sensitive to hypoxia or elevated intracellular ROS levels; indeed, during oxidative stress, ZEB1 expression is reportedly suppressed by raised ROS levels [22,23]. The damaging effects of UVA generally manifest indirectly through the generation of ROS [4], and our results show that altered ZEB1 expression in response to UVA irradiation is mediated by ROS. However, the mechanistic relationship between ROS and ZEB1 requires further investigation.

As a DNA methyltransferase, DNMT1 generally regulates gene expression by modifying the methylation of targets [38], and the identification of potential target genes in UVA-induced senescence was needed to fully characterize the UVA/ROS/ZEB1/DNMT1 axis. Firstly, *in vivo* screening revealed that p53 methylation was elevated in sun-exposed human skin compared with matched non-sun-exposed specimens. The demethylation of CpG islands within the p53 gene was confirmed *in vitro* in UVA-irradiated HDFs, and a corresponding increase in p53 expression was also observed. Although DMNT1 over-expression did not reverse the demethylation of exact CpG islands which triggered by UVA, it could cause other several CpG islands methylated instead, and reversed the increase of p53 expression induced by UVA. To our knowledge, a few studies have indirectly linked decreased DNMT1 expression with p53 promoter hypomethylationin different systems, but direct evidence of DNMT1-mediated regulation of p53 methylation has been lacking [39,40]. We present, for the first time, direct evidence that DNMT1 modulates p53 methylation; furthermore, we precisely identify the sites of methylation within the p53promoter that are affected during UVA-induced senescence. As with p53, p16 and p21 expression was up-regulated in UVA-treated HDFs; however, unlike p53, these genes exhibited no significant change in CpG methylation status. This suggests that alternative mechanisms are responsible for the UVA-induced alterations in p16 and p21 expression. For instance, ROS could regulate their expression by activating the MAPK or TGF-Smad signaling pathways [41,42].

In conclusion, we have revealed a novel pathway that is important in UVA-induced senescence. Oxidative stress and epigenetic alterations is known to be the most crucial etiology aspects of photoaging. The UVA/ROS/ZEB1/DNMT1/p53 axis we proposed created a crosslink between these two aspects, and formed a complex network promoting the pathological process of photoaging. Also, it provided multiple potential targets for interventions against UVA-induced aging.

## METHODS

### Cell culture

Primary HDFs were isolated from the circumcised foreskins of healthy human donors between 5 and12 years of age. Cells were cultured in Dulbecco’s modified Eagle’s medium (DMEM; Gibco, Grand Island, NY, USA) supplemented with 100 U/ml penicillin, 100 ng/ml streptomycin, and 10% fetal bovine serum (FBS; Gibco), at 37°C in a humidified incubator with 5% CO_2_. Before harvesting primary HDFs, written informed consent was obtained from legal guardians of donors in accordance with a protocol approved by the Clinical Research Ethics Committee at the XiangYa Hospital of Central South University in Changsha, China.

### UVA irradiation

To prevent UVA absorption by factors within the growth medium, when reached 80% confluence, HDF cells were rinsed in phosphate-buffered saline (PBS), and submerged under a thin layer of PBS prior to UVA irradiation. Cells were then irradiated three times with a UVA dose of 10 J/cm^2^ per day for 3 days, as verified with a UV light meter (Sigma, Shanghai, China), using a Philips UVA lamp (emission spectrum320 to 400 nm). Mock-irradiated cells underwent identical procedures, but were not exposed to UVA. The time interval of these three UVA irradiations is 24 hours. Following each UVA irradiation, cells were incubated in complete medium as described above.

### Western blotting

The protein was extracted from the cultured cells, and homogenized in RIPA lysis buffer (Beyotime, China) with Protease Inhibitor Cocktail (Sigma-Aldric,USA) on ice. Thirty micrograms of protein from each cell lysate were separated by SDS-PAGE using a 10% gel, and then electrophoretically transferred to a PVDF membrane (Millipore, MA, USA). Blots were probed with appropriate primary antibodies at 4°C overnight, and then incubated with an HRP-conjugated secondary antibody (Cell Signaling Technology, USA) for 1 h at room temperature. Protein bands were visualized using an Immobilon^TM^ Western HRP substrate (Millipore, USA). The antibodies used in western blot are list following: Anti-Dnmt1 antibody, Anti-p53 antibody, Anti-p21 antibody (Abcam, Cambridge, UK), Anti-p16 antibody, Anti-ZEB1 antibody Anti-β-actin antibody (SAB, USA). Image J software was used for quantitative analysis of scanning densitometric values of proteins as ratios to β-actin, which was used as a loading control.

### Senescence-associated β-galactosidase staining

The SA-β-gal activity of cells was measured using a β-galactosidase staining kit (Cell Signaling Technology, USA) according to the manufacturer’s instructions. Briefly, cells were washed in PBS, fixed at room temperature for 15 min in fixing solution, then incubated overnight at 37°C in staining solution. To calculate the relative SA-β-gal activity in each tested condition, four continuous microscopic fields were selected, and the ratio of cells exhibiting SA-β-gal activity to the total cell count was determined.

### RNA Extraction and qRT-PCR

RNA was extracted using TRIzol reagent (Invitrogen, USA) according to the manufacturer’s instructions. RNA was then reverse transcribed using the RevertAid™ First Strand cDNA Synthesis Kit (Fermentas, Canada) and quantified using a Real-Time PCR Assay kit (Thermo Scientific, USA) according to the manufacturer’s instructions. Signal detection was performed in triplicate using CFX96 Touch realtime system (Bio-Rad, USA). The reaction was performed with initial denaturation at 95 °C for 10 min, followed by 40 PCR cycles of 95 °C for 15 s and 60 °C for 60 s. Data were collected and analyzed using 2-ΔΔCt method. Values of genes were first normalized against GAPDH, and then compared to the experimental controls.

### Lentiviral transduction and siRNA transfection

HDFs were seeded at a density of 1 × 10^6^/well in 6-well plates (Corning, USA). For transduction/transfection procedures, cells were washed twice in 1 ml of PBS, then 2 ml antibiotic-free DMEM with 10% FBS was added. Next, either 2mL of pGL4.1-DNMT1 vector, pGL4.1 empty vector, DNMT1-shRNA, control-shRNA, pCWV-ZEB1 vector, or pCWV empty vector (all to 20 nM final concentration, GeneChem Company, China), or 5 ml of ZEB1-siRNA or negative control(NC)-siRNA (both to 100 µM final concentration, GenePharma, China) were added to the culture medium. Cells were then incubated at 37°C for 48 h before being harvested. The sequence of the DNMT1-shRNA oligoribonucleotide is presented in Supplementary Table 1.

### ROS measurements

The intracellular ROS level was measured using Reactive oxygen test kit (Beyotime, china). HDFs were loaded with DCFH-DA for 20 min at 37 °C in the dark and washed twice with PBS. Fluorescence was measured using Multiscan Spectrum.

### ChIP assays

In ChIP assays, approximately 1 × 10^7^ HDFs were fixed with 1% formaldehyde, then the reaction was quenched with glycine. Cells were then washed three times with PBS and harvested in ChIP lysis buffer (50 mM Tris-HCl, pH 8.0, 1% SDS, 5 mM EDTA). The lysate was sonicated to shear DNA into 400–600 bp fragments, and then cell debris was pelleted by centrifugation. The supernatant was transferred to a separate tube, and 4 volumes of ChIP dilution buffer (20 mM Tris-HCl, pH 8.0, 150 mM NaCl, 2 mM EDTA, 1% Triton X-100) was added. To precipitate specific DNA sequences, the lysate was incubated overnight at 4°C with protein-G beads (EMD Millipore, USA) and specific antibodies. Protein-G beads were washed 5 times, and then DNA was eluted in ChIP elution buffer (0.1 M NaHCO3, 1% SDS, 30 μg/ml proteinase K). The eluent was incubated at 65°C overnight, and then DNA was extracted using a DNA purification kit (Beyotime Biotechnology, China). Purified DNA was examined using a quantitative PCR kit (Bio-Rad,USA) according to the manufacturer’s instructions, and primers specific for predicted binding sites (Supplementary Table 2). Data are presented as the mean ± standard deviation (SD) of at least three independent experiments.

### Luciferase reporter assays

Luciferase reporter assays were performed using a Dual-Luciferase Reporter Assay System (Promega, Madison, WI) according to the manufacturer’s instructions. Briefly, transcription initiation sites within the DNMT1 promoter were amplified from genomic DNA by PCR, then inserted into a pGL4 control vector (Promega) using the XBA1 site immediately downstream of the luciferase stop codon. Additionally, mutant reporter genes were created using the QuikChange Lightning Multi Site-Directed Mutagenesis kit (Stratagene, USA). The primers used to clone the DNMT1 promoter and create mutants are listed in Supplementary Table 3. Reporter vectors, as well as a pCMV-HA-ZEB1 expression vector, were transfected into T293 cells using Lipofectamine 2000 reagent (Invitrogen,USA), according to the manufacturer’s instructions. Firefly and Renilla luciferase activities were measured consecutively using the Dual-Luciferase Reporter Assay System (Promega) 48 h post-transfection.

### High-throughput microfluidic PCR and next-generation bisulfite sequencing

The genomic DNA was extracted from sun and non-sun exposed skin tissue samples using the TIANampGenomic DNA Kit (TianGen Biotech, Beijing, China) according to the manufacturer’s instruction. Extracted DNA was bisulfite conversed by using an EZ DNA Methylation-Lightning^TM^kit (Zymo Research, Irvine, CA, USA). Bisulfite sequencing primers were designed for 24 senescence genes using the MethPrimer tool (http://www.urogene.org/methprimer/). High-throughput microfluidic PCR and next-generation bisulfite sequencing were then performed, as described previously [43]. Following MiSeq sequencing, paired-end read data were demultiplexed according to sample-specific barcodes, using MiSeq Reporter software with default parameters. The methylation status of CpG sites at each gene were assessed using BiQ Analyzer software version 3.0, with default parameters. For each sample, the methylation level of each gene was determined by taking the average methylation level of all of the CpG sites in that gene.

### Bisulfite sequencing PCR

The genomic DNA was extracted from HDFs underwent the treatments indicated in Fig 5B and was bisulfite conversed using the methods described above. The primers we used to amplified portions of the p53, p21 and p16 CpG islands were listed in Supplementary Table 5. The PCR conditions are as follows: 98°C for 4 min, then 40 cycles of 94°C for 45 sec, 65°C for 45 sec and 72°C for one min. A final incubation at 72°C for 8 min concluded the PCR. PCR products were verified by gel electrophoresis, and clone to pUC18-T plasmid, then screened and amplified using M13 primers. Data was analysis using QUMA methylation tool (http://quma.cdb.riken.j).

### Immunohistochemistry

Skin biopsy samples were fixed in 4% paraformaldehyde, paraffin embedded, segmented, mounted onto slides, and dehydrated according to standard protocols. Tissue immunohistochemistry staining was performed using anti-DNMT1 or anti-ZEB1 primary antibodies (1:100 dilution; Abcam, USA), and a biotinylated rabbit anti-goat IgG secondary antibody (1:200 dilution;Abcam, USA). Detection was performed using a DAB Horseradish Peroxidase Color Development kit (Beyotime Biotechnology), according to the manufacturer’s protocol. (Magnification= 400×)

### Statistical analysis

All data are representative of at least 3 independent experiments, and are expressed as means ± SD. The statistical significance of observed differences was determined by a one-way analysis of variance, followed by further analysis using the LSD (least significant difference) test. P < 0.05 was considered statistically significant.

## Supplementary Material

Supplementary File
