# Supplementary material for: Ultraviolet A irradiation induces senescence in human dermal fibroblasts by down-regulating DNMT1 via ZEB1
Source: Aging (Albany NY). 2018 Feb 16;10(2):212–28. doi: 10.18632/aging.101383 (PMC5842848; doi:10.18632/aging.101383)
Supplement: Supplementary File [file aging-10-101383-s001.pdf]

SUPPLEMENTARY MATERIAL

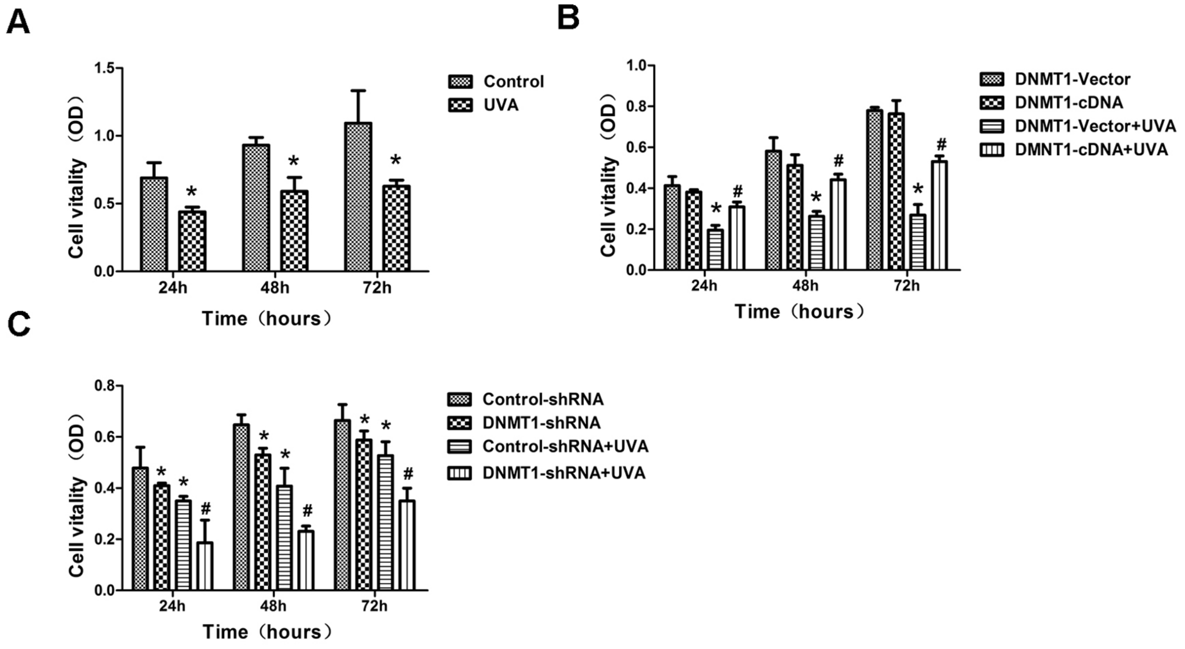

Supplementary Figure 1. DNMT1 attenuates UVA-induced decrease of cell vitality in HDFs. (A, B, C,) HDFs were transfected with or without the indicated expressing lentivirus, then cell vitalities were determined by MTT assay following UVA irradiation (n = 3). * vs Control, DNMT1-vector or control-shRNA, P< 0.05, # vs DNMT1-vector+UVA or control-shRNA+UVA, P< 0.05

Supplementary Table 1. The sequences of DNMT1-shRNA oligoribonucleotides.

NO.	5’	STEM	Loop	STEM	3’
DNMT1-RNAi(19531-1)-a	Ccgg	aaCGGTGCTCATGCTTACAAC	CTCGAG	GTTGTAAGCATGAGCACCGTT	TTTTTg
DNMT1-RNAi(19531-1)-b	aattcaaaaa	aaCGGTGCTCATGCTTACAAC	CTCGAG	GTTGTAAGCATGAGCACCGTT	

Supplementary Table 2. The primers for amplifying predicted binding sites in ChIP assays.

binding si te 1	Forward:5'- TTCTCGCTGCTTTATCCCCA -3'<br>Reverse: 5'- CATTCATTCATTCATTCTTT -3'
binding site 2	Forward:5'- GGTGCAATTACCCCGTTTTA -3'<br>Reverse: 5'- CTACGGCTCAGCCTCTGTGT -3'
binding site 3	Forward:5'- CTCCTAACCTCAAGCGATCC -3'<br>Reverse: 5'- TCCCAGTGCTTTGAGAGGCC -3'
binding site 4	Forward:5'- CTCCCAAGTAGCTAGGATTA -3'<br>Reverse: 5'- GATTTGCTGGGCATGGTGAC -3'

Supplementary Table 3. The primers that used for cloning DNMT1 promoter and deletion mutation of predicated binding sites.

DNMT1 promoter	Forward:5'-CAGCCTACACTGCCAGGG-3'<br>Reverse: 5'-GTACGCGCCGGCATCTCG-3'
DNMT1 m1	Forward:5'- CTCCCAAGTAGCTAGGATTAGTCACCATGCCCAGCAAATC -3'<br>Reverse: 5'- GATTTGCTGGGCATGGTGACTAATCCTAGCTACTTGGGAG -3'
DNMT1 m2	Forward:5'- GATTTGCTGGGCATGGTGACTAATCCTAGCTACTTGGGAG -3'<br>Reverse: 5'- TCCCAGTGCTTTGAGAGGCCGGATCGCTTGAGGTTAGGAG -3'
DNMT1 m3	Forward:5'- GGTGCAATTACCCCGTTTTAACACAGAGGCTGAGCCGTAG-3'<br>Reverse: 5'- CTACGGCTCAGCCTCTGTGTTAAAACGGGGTAATTGCACC -3'
DNMT1 m4	Forward:5'- TTCTCGCTGCTTTATCCCCAAAAGAATGAATGAATGAATG-3'<br>Reverse: 5'- CATTCATTCATTCATTCTTTTGGGGATAAAGCAGCGAGAA-3'

Supplementary Table 4. The average mathylation of 24 senescent-associated genes.

The abbreviation of genes	Average Mathylation				
	Non-sun exposed		sun exposed		P value
	Mean	SD	Mean	SD	
**pRb**	0.06275	0.04303	0.0100	0.002273	0.2667
**ATM**	0.0285	0.001190	0.04333	0.01011	0.1441
**NF-κB1**	0.01975	0.01020	0.008000	0.001000	0.3761
**Sirt1**	0.1560	0.1460	0.0160	0.003512	0.3920
**NANOG**	0.2390	0.005307	0.2393	0.005121	0.9741
**SP1**	0.8185	0.07694	0.8935	0.04056	0.4217
**SOX2**	0.0545	0.03053	0.0860	0.06502	0.6499
**VDR**	NA	NA	NA	NA	NA
**ZEB1**	NA	NA	NA	NA	NA

**ZEB2**	0.9240	0.01251	0.9300	0.01002	0.7387
**PTEN**	0.03475	0.01026	0.0440	0.02301	0.7012
**Foxd3**	0.3925	0.1482	0.4863	0.1204	0.6621
**Dnmt3a**	0.8033	0.08352	0.7217	0.09033	0.5409
**Dnmt3b**	0.9177	0.08233	0.8405	0.1595	0.6616
**p53**	0.6860	0.04623	0.4945	0.06067	0.0459
**p21**	0.01567	0.007688	0.01033	0.002028	0.5391
**p16**	0.1858	0.07477	0.1985	0.07331	0.9071
**LEF1**	0.03525	0.004661	0.07333	0.03405	0.2463
**UTF1**	0.0485	0.01850	0.0300	0.01384	0.4539
**TERT**	0.7320	0.05921	0.8125	0.03422	0.2639
**SFRP2**	0.09975	0.05778	0.04275	0.03580	0.4338
**KIT**	0.03775	0.03042	0.01225	0.005072	0.4400
**GRB7**	0.3618	0.1162	0.1597	0.06757	0.2322
**CTNNB1**	0.04075	0.0008539	NA	NA	NA

NA: Not Applicable

Supplementary Table 5. The primers for amplifying p53, p21, p16 GpC island regions.

p53	Forward:5'- GAGTAGGTAGAAGATTTTYGGGAG -3'<br>Reverse: 5'- AAACCTACTACRCCCTCTACAAAC -3'
p21	Forward:5'- GGAGTGTAGGTGGTATGATTTTAG -3'<br>Reverse: 5'- TTCCTAACATCACAAATCTAAAATAC -3'
p16	Forward:5'- AGTTTAGAAAGGATYGGTGATGTG -3'<br>Reverse: 5'- AAACAAACACCRAATCCTTTATATC -3'

SUPPLEMENTARY METHODS

3-(4,5-Dimethylthiazol-2-yl)-2,5-diphenyltetrazolium bromide (MTT) assay

HDFs cells were planted on 96-well plates at a density of 4000 cells per well in triplicate, and exposed to UVA or not. After additional incubation for 24, 48, or 72h, 20ml of MTT stock solution (5 mg/mL MTT reagent diluted in PBS; Sigma-Aldrich, USA) was added to each well. The plates were further incubated for 4 h at 37°C and 5% CO_2 in the dark. The supernatant was carefully removed without disturbing the sediment and 150 μL dimethyl sulfoxide (Sigma-Aldrich, USA) was added to the wells to dissolve the purple formazan crystals. The absorbance at 490 nm was obtained from a micro-plate reader (BioRad). All experiments were performed in triplicate, and the data presented represent the means of 3 independent experiments ± SD.
